# Comparative dynamics of Japanese encephalitis virus adaptation in porcine macrophages and insect cells

**DOI:** 10.1186/s12985-026-03231-0

**Published:** 2026-06-27

**Authors:** Andrea Marti, Alexander Nater, Jenny Pego Magalhaes, Noelle Donzé, Markus Gerber, Lea Almeida, Matthias Liniger, Sandra Chenaux, Nicolas Ruggli, Fadi G. Alnaji, Marco Vignuzzi, Obdulio Garcia-Nicolas, Artur Summerfield

**Affiliations:** 1 Institute of Virology and Immunology IVI, Mittelhäusern, Switzerland; 2 Department of Infectious Diseases and Pathobiology, Vetsuisse Faculty, University of Bern, Bern, Switzerland; 3 Graduate School for Cellular and Biomedical Sciences, University of Bern, Bern, Switzerland; 4 Interfaculty Bioinformatics Unit (IBU) and Swiss, Institute of Bioinformatics (SIB), University of Bern, Bern, Switzerland; 5 A*STAR Infectious Diseases Labs (A*STAR ID Labs), Agency for Science, Technology and Research (A*STAR), Singapore, Singapore; 6 Department of Microbiology and Immunology, Infectious Diseases Translational Research Programme, Yong Loo Lin School of Medicine, National University of Singapore, Singapore, Singapore

**Keywords:** JEV, *Orthoflavivirus*, Pigs, Mosquitos, Macrophages, Fitness adaptations

## Abstract

**Background:**

Japanese encephalitis virus (JEV) is a zoonotic mosquito-borne *Orthoflavivirus* that circulates primarily in birds and pigs. Previous observations of vector-free transmission between pigs indicates the possibility of single-host cycling in swine. Therefore, the aim of this work was to investigate the evolutionary pressure of single host cycling using a relevant primary cell culture model.

**Methods:**

To investigate whether such single-host cycles affect viral infectivity, fitness and genomic adaptations, two strains and a reverse genetic cDNA-derived clone of JEV were serially passaged 12 times in primary porcine monocyte-derived macrophages (MDMs), in *Aedes albopictus*-derived C6/36 cells, and alternately between both cell types. Next-generation sequencing analysis was used to identify selected single nucleotide variants (SNVs) and haplotypes. Phenotype-to-genotype connections were confirmed using reverse genetics.

**Results:**

For all viruses, serial passaging in MDMs - but not in C6/36 cells - led to a rapid increase in relative infectivity toward MDMs, accompanied by reduced plaque sizes in porcine endothelial cells. In contrast to C6/36 cells, MDM imposed a strong selective pressure, rapidly favoring selection of many SNVs and viral haplotypes. In addition, we identified a dominant selection of mutants with glutamic acid to lysine substitutions at positions 49 or 138 in the E protein, which explained the small plaque phenotype and caused viral sensitivity to heparin-mediated inhibition of attachment, indicating enhanced virus binding to glycosaminoglycans (GAG). The E138K mutant also explained the increased relative infectivity for MDM.

**Conclusion:**

This work demonstrates a high evolutionary pressure on JEV in MDM causing rapid selections of minor haplotypes. Furthermore, the efficient selection of E49K and E138K SNV, which were responsible for the phenotype, are likely caused by a selective pressure for GAG binding, observed *in vitro* with other mammalian cells.

**Supplementary Information:**

The online version contains supplementary material available at https://doi.org/10.1186/s12985-026-03231-0.

## Background

Japanese encephalitis virus (JEV) is a zoonotic mosquito-borne Flavivirus that can cause severe encephalitis in humans, as well as reproductive failure in pigs. The virus is endemic in South-East Asia and Oceania [38],van den [39]. While in humans, viremia is too low to infect mosquito vectors, infection of pigs, sheep and birds causes sufficient viremia for infection of biting mosquitoes, and thereby continued cycling of JEV between vertebrate and insect hosts. This cycling is maintaining the virus in the environment and thereby the basis for human infection [29, 42].

Although vector-borne transmissions are the main route of infection for JEV, vector-free direct transmission in pigs has been described experimentally [22, 32]. A role of such a direct transmission in the field is supported by the observation that circulation of JEV in pig farms did not always correlate to the prevalence of infected vectors [8, 27], and by mathematical modeling based on a field study in Cambodia [13].

The error prone viral RNA replication, as well as the quasispecies nature of RNA viruses enables a rapid adaptation to new environments [12, 41], such as single host cycling. Indeed, passaging of JEV in pigs was found to cause fitness adaptation that were associated with enhanced virulence [22]. Accordingly, the present study was initiated to model how a switch from dual- to single-host cycling might impact viral adaptation to cells of such hosts. To this end, JEV was passaged 12 times in insect C6/36 cells, in primary porcine macrophages and in the two cell types in an alternating manner. The alternating passaging was used to model the dual-host transmission cycles. The C6/36 passaging was used as a model of vertical transmission in mosquitos, while the macrophage passaging served to model direct transmission between pigs. We employed primary macrophages, which not only efficiently support JEV replication [3, 15, 45] but also represent a cell type of key importance to the innate immune system, thereby providing a relevant model to mimic viral pressure [23]. To identify possible strain differences, the passages were performed using two natural isolates and a cDNA-derived clone of JEV. The passaged viruses were characterized phenotypically and genetically, followed by linking phenotype and genotype. We show that porcine macrophages, unlike C6/36 cells, exert a strong influence on viral adaptation while simultaneously driving the rapid emergence of glycosaminoglycan-binding variants.

## Methods

### Cells

The *Aedes albopictus* C6/36 (ATCC) cell line was cultured at 28°C and 5% CO_2_ using minimal essential medium (MEM) supplemented with non-essential amino acid solution, sodium pyruvate and 10% fetal bovine serum (FBS, medium and all supplements from Thermo Fisher Scientific). Porcine monocyte-derived macrophages (MDMs) were differentiated from peripheral blood mononuclear cells (PBMCs) as previously described [33]. Porcine blood was collected from specific pathogen-free (SPF) Swiss Large White pigs bred at the IVI in Mittelhäusern, Switzerland. The protocols were reviewed by the committee on animal experiments of the canton of Bern, Switzerland, and authorized by the cantonal veterinary authority under the license number BE127/2020. PBMCs were isolated from the citrated blood by density gradient centrifugation over Ficoll-Paque (GE HealthCare). Monocytes were sorted as CD172a^+^ cells using magnetic cell sorting. The cells were differentiated into MDMs by incubation for 4 d at 39°C and 5% CO_2_ in Dulbecco’s modified eagle medium (DMEM, Thermo Fisher Scientific) supplemented with 10% FBS and 20 U/ml porcine M-CSF (produced in-house) [33]. PEDSV.15 cells (CLS 305211, kindly provided by Jörg Seebach, University of Geneva, Switzerland) [36] were cultured in DMEM containing GlutaMAX, non-essential amino acids, 1 mM sodium pyruvate (all from Thermo Fisher Scientific), 7% horse serum (Biowest) and 2% porcine serum (Sigma-Aldrich, Merck) at 37°C and 5% CO_2_. Vero cells (ATCC; CCL-81) were cultured in DMEM supplemented with 10% FBS and non-essential amino acids. During viral inoculation, medium without supplements was used (referred to as inoculation medium). During infection (referred to as infection medium), the serum concentration in the medium was reduced to 2%.

### Viruses isolates and JEV reverse genetics

JEV Laos (CNS769_Laos_2009; kindly provided by Remi Charrel, Aix-Marseille Université, Marseille, France) is a genotype I-b virus that was isolated from the cerebrospinal fluid of a human patient in Laos [5]. JEV Cambodia (CO81; gene bank accession number: KY927816; European Virus Archive global), a genotype I-a virus, was extracted from the cerebrospinal fluid of a fatal encephalitis case [14]. We also applied a cDNA-derived virus of JEV Laos (termed JEV Laos RG) which was generated as follows. The full-length cDNA plasmid encoding JEV Laos (JEV/CNS769/Laos/2009, GenBank accession number KC196115.1) was constructed based on the low-copy number backbone plasmid p4JEV-MCS. Briefly, p4JEV-MCS was obtained after ligation of the annealed oligonucleotides pACJR1_MCS_F (5’-P-GATCTGCGGCCGCGGTACCGAGCTCATCCCGGGACTGTCCAG TACTGCAATCGATTC) and pACJR1_MCS_R (5’-P-GGCCGAATCGATTGCAGTACTG GACAGTCCCGGGATGAGCTCGGTACCGCGGCCGCA) in the *Not*I/*Bgl*II-linearized plasmid vector pACJR1 [30] to contain unique *Not*I, *Xma*I and *Cla*I restriction sites. The constructs pJEV-Laos-5′h and pJEV-Laos-3′h (synthesized at GenScript, Leiden, the Netherlands) contain the 5′ and 3′ halves of the JEV Laos genome, separated be an internal *Xma*I restriction site. In addition, pJEV-Laos-5′h consists of a minimal T7 promoter (TAATACGACTCACTATAG) for *in vitro* transcription of the viral RNA and pJEV-Laos-3′ is flanked by a hepatitis delta virus ribozyme sequence (HDVr, GGGTCGGCATGGCATCTCCA CCTCCTCGCGGTCCGACCTGGGCTACTTCGGTAGGCTAAGGGAGAAGA) at the 3’-end for the generation of authentic 3’-ends of the viral RNA transcripts. The unique *Xma*I and *Cla*I sites served to assemble the full-length pJEV-Laos from pJEV-Laos-5′h and pJEV-Laos-3′h. Capped T7-vRNA was generated from *Cla*I linearized pJEV-Laos using the mMESSAGE mMACHINE T7 Transcription Kit (Thermo Fisher Scientific) and was purified using Illustra MicroSpin S-400 HR columns (GE Healthcare). For electroporation, 8 × 10^6^ Vero cells in 0.4 ml of PBS were mixed with 1 μg of purified RNA transcript and transferred to a 0.2-cm electroporation cuvette (BTX, Holliston, MA, USA). Electroporations were performed using an ECM 830 electroporator (BTX, Holliston, MA, USA) with the following settings: 980 V, two pulses of 100 µs pulse length at a 0.1 s interval. The cells were transferred to a T75 cell culture flask and incubated for 72 h. To produce virus master stocks, monolayers of C6/36 cells were infected (MOI 0.1) and virus containing cell culture supernatants were harvested after three days of incubation. To rescue JEV with specific E49K and E138K deviations, the plasmids pJEV-RG-E-E49K and pJEV-RG-E-E138K were constructed by site-directed mutagenesis of pJEV-Laos. Briefly, a 1.8 kb *Not*I/*Xho*I fragment of pJEV-Laos, encompassing the E protein coding region, was subcloned into pcDNA6-V5-His B (Thermo Fisher Scientific) serving as a template for inverse PCRs using Phusion Hot Start II DNA Polymerase (Thermo Fisher Scientific) and specific 5’-phosphorylated oligonucleotides (E49K-mut-F, CATGATCAACATTAAAGCTAGCCAAC and E49K-mut-R, CGGACATCTAGTGTTGGCTTG or E138K-mut-F, CATCAAGTACAAGGTTGGCATATTC and E138K-mut-R, TTCTCTGGTTGGATCATTCTTCC) (Microsynth, Switzerland). The plasmids pcDNA6-JEV-E49K-XN and pcDNA6-JEV-E138K-XN were obtained after re-ligation of the corresponding PCR product. The full-length plasmids pJEV-E49K and pJEV-E138K were constructed by the substitution of the pJEV-Laos *Not*I/*Xho*I fragment with corresponding fragments from pcDNA6-JEV-E49K-XN and pcDNA6-JEV-E138K-XN, respectively. Sanger DNA sequencing was used to verify the identity of the newly obtained constructs.

### *In vitro* passaging of JEV

JEV was passaged on insect C6/36 cells, on porcine MDMs and in an alternating manner between C6/36 and MDMs to generate JEV Px-C6/36, Px-MDM and Px-Alt, respectively. Briefly, the three lines of passages (A, B and C) were performed with each of the viruses. For P1, confluent cells in a 6-well plate were inoculated with an MOI of 0.1 TCID_50_/cell. After 1.5 h, at cell-type defined temperatures and 5% CO_2_, the inoculum was removed. The cells were washed three times with D-PBS (Thermo Scientific Fisher), and incubated in infection medium for 48 h. The harvested supernatant was centrifuged at 1000 x g for 7 min at 4°C and stored at -80°C. Subsequent passages were performed with the same methodology using 50µl of the previous passage for the inoculation of new confluent cells until P10. From P10, master stocks (termed P11) were prepared by infecting a T25 flask at an MOI of 0.05 TCID_50_/cell for 72 h. For the working stocks (termed P12), a T75 flask was infected with 1 ml of P11 and incubated for 48 h. P12 was used for the viral growth kinetics, while the individual passages were used in the relative infectivity and the focus forming assay. Reverse passaging of the JEV P10-MDM on C6/36 cells was performed as described above, starting with 50µl of P10-MDM viruses for five additional passages on C6/36 cells.

### Virus titrations

Virus stocks and passages were titrated on indicated cells and expressed as TCID_50_/ml. To this end, confluent cells in a microtiter plate were infected with 10-fold dilutions of virus samples, and incubated for 72 h at the cell type-specific temperature and 5%CO_2_. At 72 h post infection (hpi) the cells were washed once with PBS and fixed with 4% (w/v) paraformaldehyde (Polysciences) in dissolved in PBS. For immunoperoxidase staining of JEV E protein, the cells were incubated with the mouse anti-Flavivirus antibody (ATCC, HB-112, D1-4G2-4-15, hybridoma supernatant), diluted 1/20 in 0.3% saponin (HuberLab) at 37°C. After 30 min, the cells were washed three times and incubated with a 1/250 dilution of rabbit anti-mouse HRP antibody (Dako, Agilent Technology) in 0.3% saponin. Following 30 min of incubation and the three washing steps, AEC solution [50 mM Na-Acetate (Merk); 5% v/v 3-Amino-9-ethylcarbazol (Sigma) in N,N-Dimethylformamid (Sigma) and 0.05% v/v H_2_O_2_], was added and the plates were incubated for 30 min at room temperature. The stained plate was washed and covered with tap water for the readout. The titres were calculated as described [31].

### Focus-forming assay

Tenfold serial dilutions of virus preparations were used to inoculate triplicate cultures of confluent C6/36 and PEDSV.15 cells in a 24-well plate format. For infections, the cultures were incubated for 1.5 h at the cell-dependent temperatures. After the incubation, the cells were washed with D-PBS and covered with semifluid infection media containing 1% of methylcellulose (Merck). The viscous overlay was washed away at 72 hpi with PBS, and the cells were fixed prior to E-protein staining as described above. Stained viral foci were pictured and counted by Immunospot (Cellular Technology LTD). The foci size diameter was determined using Image J software [34].

### Inhibition of attachment by heparin

To inform on possible selection of JEV variants with increased binding to glycosaminoglycans (GAGs), we tested the sensitivity of blocking JEV attachment to Vero cells using heparin. Approximately 200’000 TCID_50_ of each virus (diluted in GMEM-BHK21 (Gibco 21710-025) + 2% FBS) was mixed with heparin diluted in PBS to a final concentration of 250U/ml (Bichsel 140 0 020, Switzerland) in a total volume of in 200 μl (100 μl virus-dilution + 100 μl heparin-dilution) for 30 min at 37°C, and then added to a confluent monolayer of Vero cells cultured in 24-well plates in GMEM (Thermofisher) supplemented with 2% FBS. After 2 h of incubation at 4°C, the plates were washed three times with 1 ml PBS (4°C), and 500 μl Trizol was added for RNA extraction and viral genome copy quantification. The inhibition percentage was calculated relative to controls without heparin (only PBS).

### Viral RNA quantification by RT-qPCR

Viral RNA extraction was performed using the NucleoMag VET kit (Macherey Nagel) with the extraction robot Kingfisher Flex (Thermo Scientific) following the manufacture’s guidelines. For the RT-qPCR the AgPath-ID One Step RT-qPCR kit (Thermo Fisher Scientific) was used with the forward primer 5’-ATC TGA CAA CGG AAG GTG GG-3’, the reverse primer 5’-TGG CCT GAC GTT GGT CTT TC-3’ and the probe 5’ FAM- AGG RCC CTG CTC ACC GGA AGT- TAMRA-3’. The quantity of viral RNA was assessed relative to an RNA standard (in house produced, as previously described [22]) using dilutions of 10^8^ to 10^2^ copies/µl. The cut-off was set at 10^2^ copies/µl. The plates were analyzed by the 7500 Applied Biosystems Real-time PCR machine (Thermo Fisher Scientific).

### Viral genome analysis

For next generation sequencing, viral RNA from the passage 1, 3, 5, 7, 10 and 12 of each line of JEV was diluted 1:3 in Trizol reagent (Thermo Fisher Scientific). A 49:1 mixture of Chloroform (Sigma) and Isoamylalcohol (Sigma) was added, leading to a phase separation. The aqueous phase was mixed 1:1 with 75% ethanol (Merck) and loaded on a NucleoSpin RNA column. The RNA was extracted following the guideline of the Nucleospin RNA extraction Kit (Macherey Nagel). The cDNA libraries were generated using the NEBNext Ultra II RNA Library Prep kit for Illumina (New England Biolabs). The samples were purified by the NucleoMag NGS Clean-up (Machery Nagel) and quantified by Qubit dsDNA HS Assay (Invitrogen) measuring fluorescence using the TECAN machine (infinite M200 Pro). The samples were sequenced with the NextSeq500/550 v2.5 kit (Ilumina) on the NextSeq500 instrument (Ilumina) with single reads and 151 cycles.

The data was analysed as previously described [22] with some modifications. The sequencing quality was assessed using Illumina Sequencing Analysis Viewer (Illumina version 2.4.7) and all base call files were demultiplexed and converted into FASTQ files using Illumina bcl2fastq conversion software v2.20. Quality control of raw sequencing reads used FastQC v0.11.9 [4]. Read pairs were classified using Kraken2 v2.1.2 [44] by exact k-mer matching to a custom database consisting of multiple host and potential contaminant genomes, including GRCh38 (*Homo sapiens*), Sscrofa11.1 (*Sus scrofa*), GCF_006496715.1 (*Aedes albopictus*), and GCF_001876365.2 (C6/36), as well as JEV sequences KY927816, KC196115, and EF571853. We extracted all read pairs classified as being derived from JEV for further analysis.

Reference sequences of the four strains were assembled from pre-processed and filtered reads of the P0 samples using SPaDES v3.15.5 [19] in the ‘rnaviral’ mode. The resulting reference genomes were manually curated and GTF files of gene annotations were created based on alignments of protein-coding sequences to the assembled genomes. Reads were mapped to the corresponding strain-specific reference genome using BWA mem v0.7.17 [19] and SAM files were converted to BAM format with SAMtools v1.17 [20]. Single-nucleotide variants (SNV) were called per sample using LoFreq v2.1.5 [43] without applying default filters for coverage or strand bias. Genome-wide mapping statistics were obtained with the flagstat command of SAMtools v1.17 [20] and Mosdepth v0.3.3 [28]. Analysis of the site-wise sequencing depth for each sample was performed with the depth command of SAMtools v1.17 [20]. Variant effects and sequence statistics were calculated with SNPGenie v1.0 [25]. Allele frequency trajectories and genetic diversity statistics along the passages were generated with custom Python scripts using the sample-wise VCF files and filtering for a minimum site-wise sequencing depth of 100 based on the output of SAMtools depth. Networks of pairwise allele frequency correlations between variant sites across passages were generated with Cytoscape v3.10.1 [37]. All analyses were run in R version 4.2.1 (2022-06-23).

### Statistical analyses

Statistical analyses were performed using Graph Pad Prism 8.0 and indicated in the Figure legends (GraphPad Software, La Jolla, USA). Significance was determined by the P values lower than 0.05, depicting *p<0.05, **p<0.01; ***p<0.001; **** p<0.0001.

## Results

### Rapid adaptation of JEV towards increased infectivity for MDMs following passaging in MDMs

The JEV strains Cambodia, Laos and the cDNA-derived Laos RG were passaged ten times in triplicate to generate three separated lines of passaging (called A, B and C) in C6/36, MDM or by alternating between the two cell types. To identify potential adaptive changes after serial passages of the viruses, the relative infectivity of the passaged viruses was determined by calculating the ratios of the titers obtained in MDMs *versus* C6/36. For each passage the relative infectivity was statistically compared to P0.

**Fig. 1:**
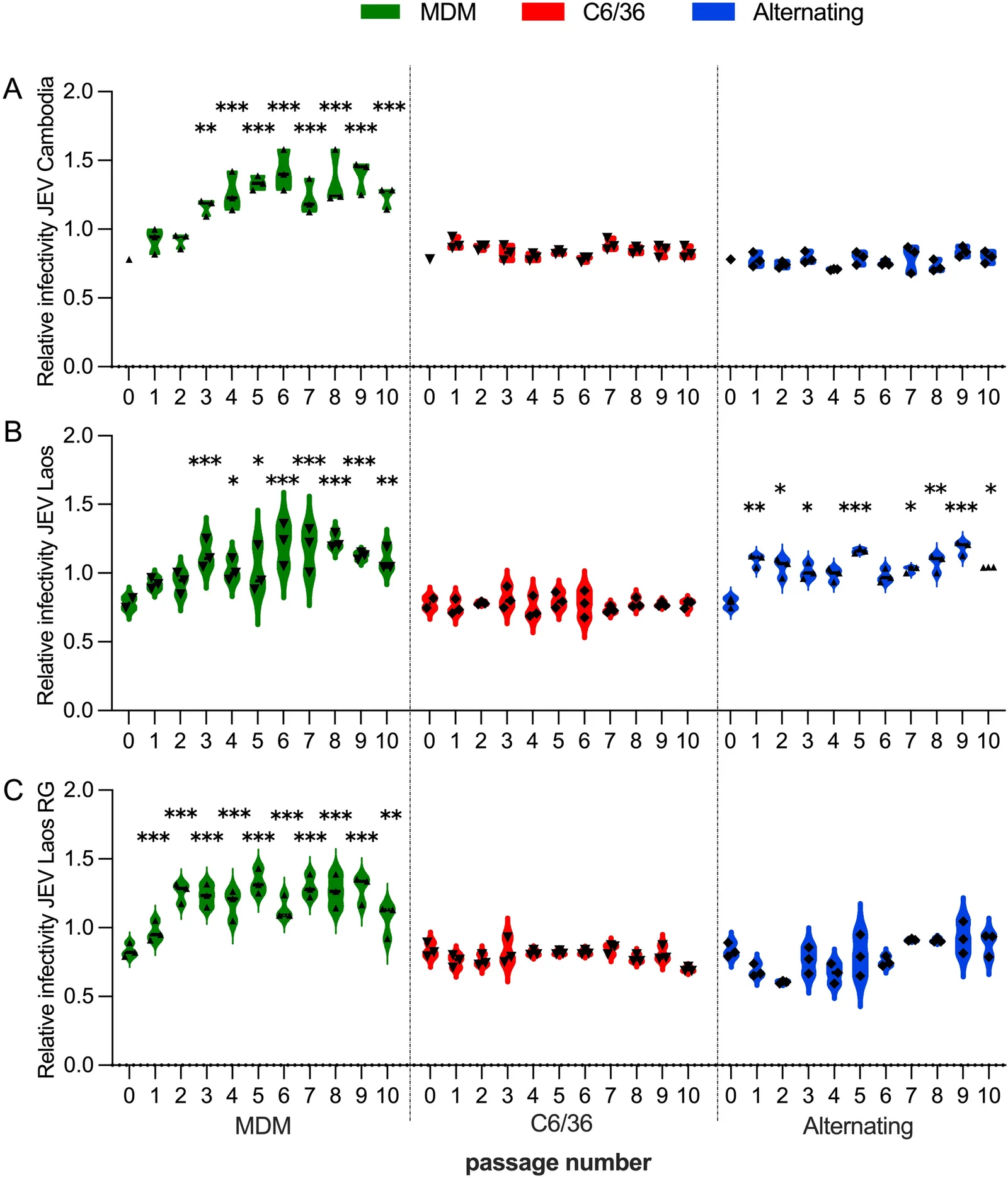
Changes in relative infectivity following passaging of JEV. The JEV strains Cambodia (**A**), Laos (**B**) and the cDNA-derived clone JEV Laos RG (**C**) were passaged 10 times in MDM (green symbols), C6/36 (red symbols) or in both cell types in an alternating manner (blue symbols). All passages (P) were characterized by titration on MDM and C6/36 to determine the relative infectivity calculated as the ratio of the log10 TCID_50_ titres on MDM and C6/36. Significant differences compared to the P0 values were determined by Tukey’s multiple comparison test (*p<0.05, **p<0.01, ***p<0.001 and ****p<0.0001)

Passaging of JEV Cambodia in MDM caused a significant increase in relative infectivity as early as P3. In contrast, following alternating or C6/36 passaging, no adaptations were found (Fig. 1A). Also, the passaging of JEV Laos in MDM resulted in increased relative infectivity for MDM after 3 passages. For this virus strain, also the alternating passaging caused a significant increase as early as P1. As with JEV Cambodia, following passaging in C6/36, no such adaptation was observable (Fig. 1B). With JEV Laos RG, the increase in relative infectivity was only found following passaging in MDMs already in P1 (Fig. 1C).

### Rapid adaptation of JEV to a small focus size phenotype following passaging in MDMs

**Fig. 2:**
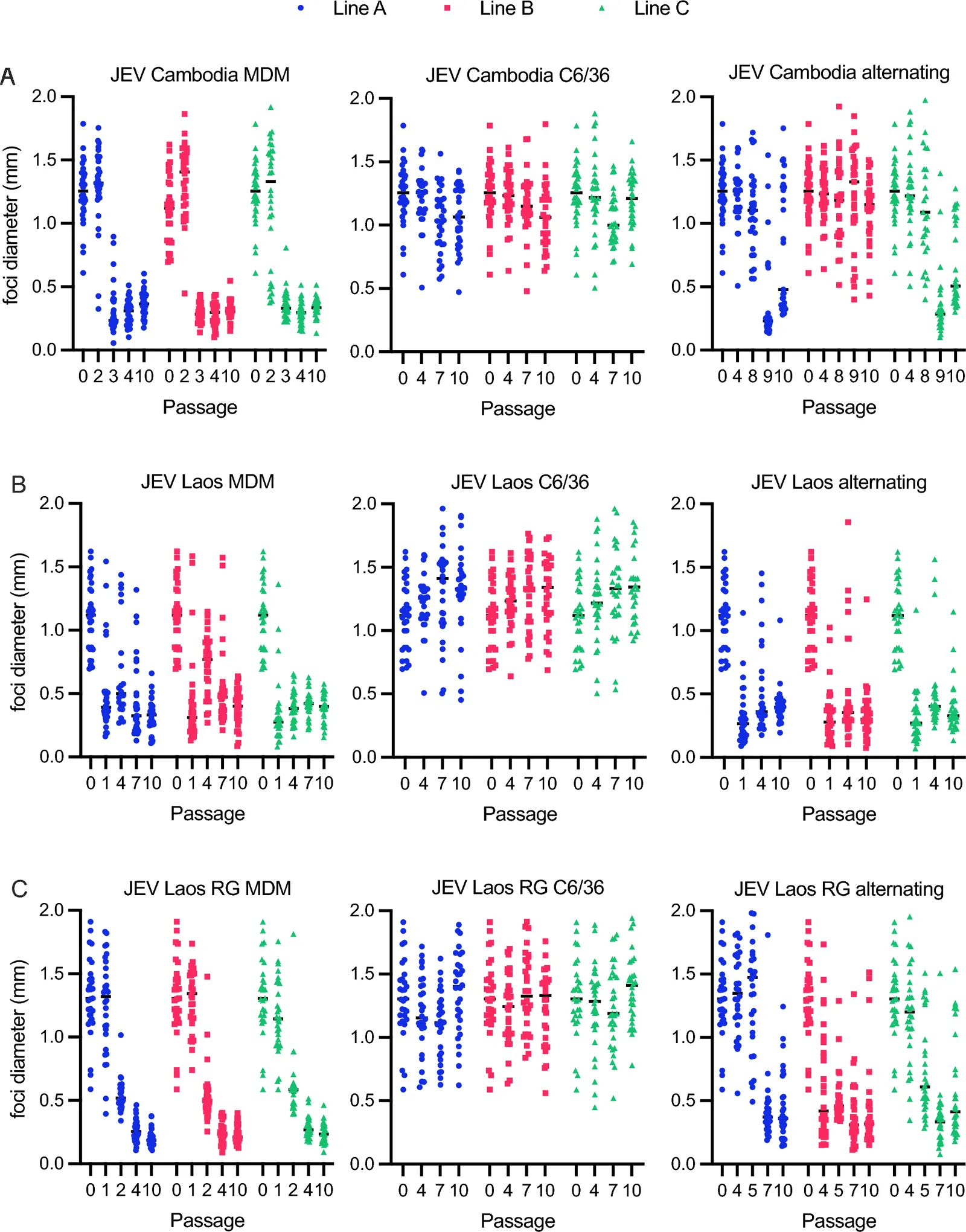
Changes in foci sizes following passaging of JEV. JEV Cambodia (**A**), JEV Laos (**B**) and JEV Laos RG (**C**) were passaged 10 times in MDM, C6/36, or in both cell types in an alternating manner (left to right). At the indicated passages the viruses were characterized by focus-forming assays using PEDSV.15 cells. Line A is depicted in blue, line B in red and line C in green. Statistical significances of changes in plaque sizes are found in Supplementary Table 1

As an alternative readout for phenotypic adaptation, we employed a focus-forming assay using monolayers of PEDSV.15 cells, a porcine aortic endothelial cell line. MDMs could not be used in this assay as they do not form confluent monolayers. Passaging of JEV Cambodia in MDM caused a foci size reduction affecting all infectious foci by P4. This selection was not observed in C6/36, while the alternating passaging caused a mixed phenotype in lines A and C with small and large foci. In line B, no selection was observed (Fig. 2A). For JEV Laos, a selection for small foci was also induced by passaging in MDM. In lines A and B, solely small foci were found after ten passages, and in line C after four passages. Also, the alternating passaging of JEV Laos caused selection of small foci but a mixed phenotype persisted during passaging with one or more large foci observable in P10. In contrast, all plaques remained large following C6/36 passaging (Fig. 2B). For JEV Laos RG, we observed a similar phenotypic change as for the parent JEV Laos. The emergence of a pure small focus phenotype was observed after four passages in MDM in all three lines. Alternating passaging again caused a mixed phenotype with mostly small foci in P7 and P10. Again, the focus size after passaging in the insect cells remained mostly unchanged (Fig. 2C). Of note, we also performed the focus size assays in C6/36 and found a reduction of focus size following MDM passaging. However, the foci sizes in C6/36 were generally smaller when compared to PEDSV.15 cells, explaining why their size reduction was not as visible with C6/36 cells (Supplementary Figure 1).

We next investigated the stability of the small-focus phenotype induced by passaging in MDM. To this end, the P10 of the MDM lines A, B and C were passaged five times in C6/36 cells, and the focus sizes were analyzed again. For all three JEV, the small-focus phenotype was maintained during passaging in C6/36 (Supplementary Figure 2).

Taken together, these data demonstrate that passaging in MDM but not in C6/36 cells cause a strong selection pressure for small foci sizes. This evolutionally pressure was also present during alternating passaging, albeit at reduced levels.

### Correlation of increased relative infectivity and small foci sizes

**Fig. 3:**
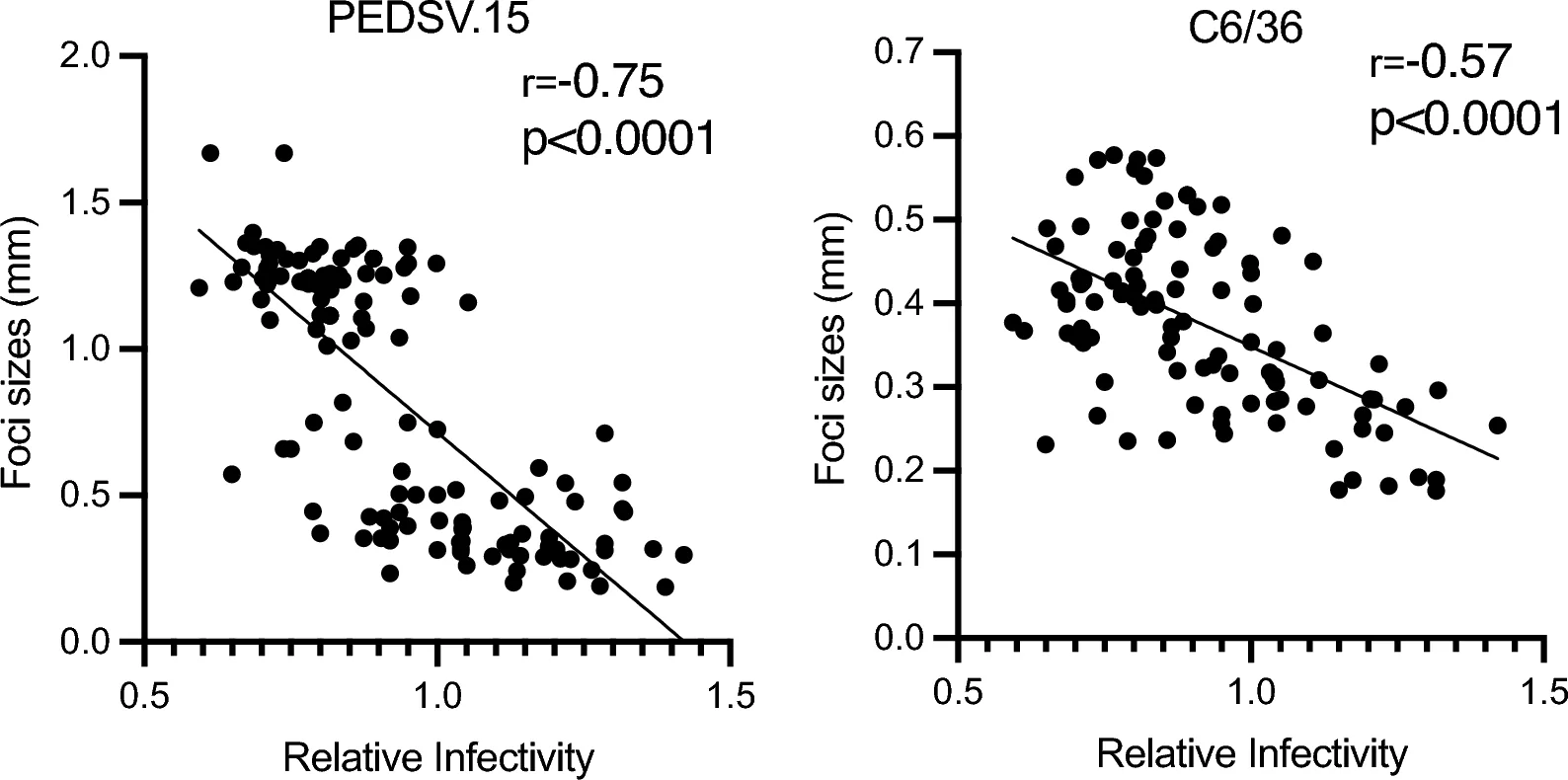
Pearson correlation between relative infectivity and foci sizes**.** The Pearson correlation of relative infectivity values from Fig. 1 with the foci sizes from Fig. 2 (PEDSV.15 cells) and Supplementary Figure 1 (C6/36 cells) were calculated and presented as graphs. The correlation coefficient r and the p values are indicated in the upper left corner of the graphs

To determine a possible association between the increased infectivity for MDM and small foci sizes, we performed correlation analyses of the two parameters for all data acquired. Figure 3 demonstrates a high level of correlation when PEDSV15 or C6/36 were used for the foci size assay indicating that the two observations might be linked to the same genetic adaptations.

### Impact of passaging on nucleotide changes

**Fig. 4:**
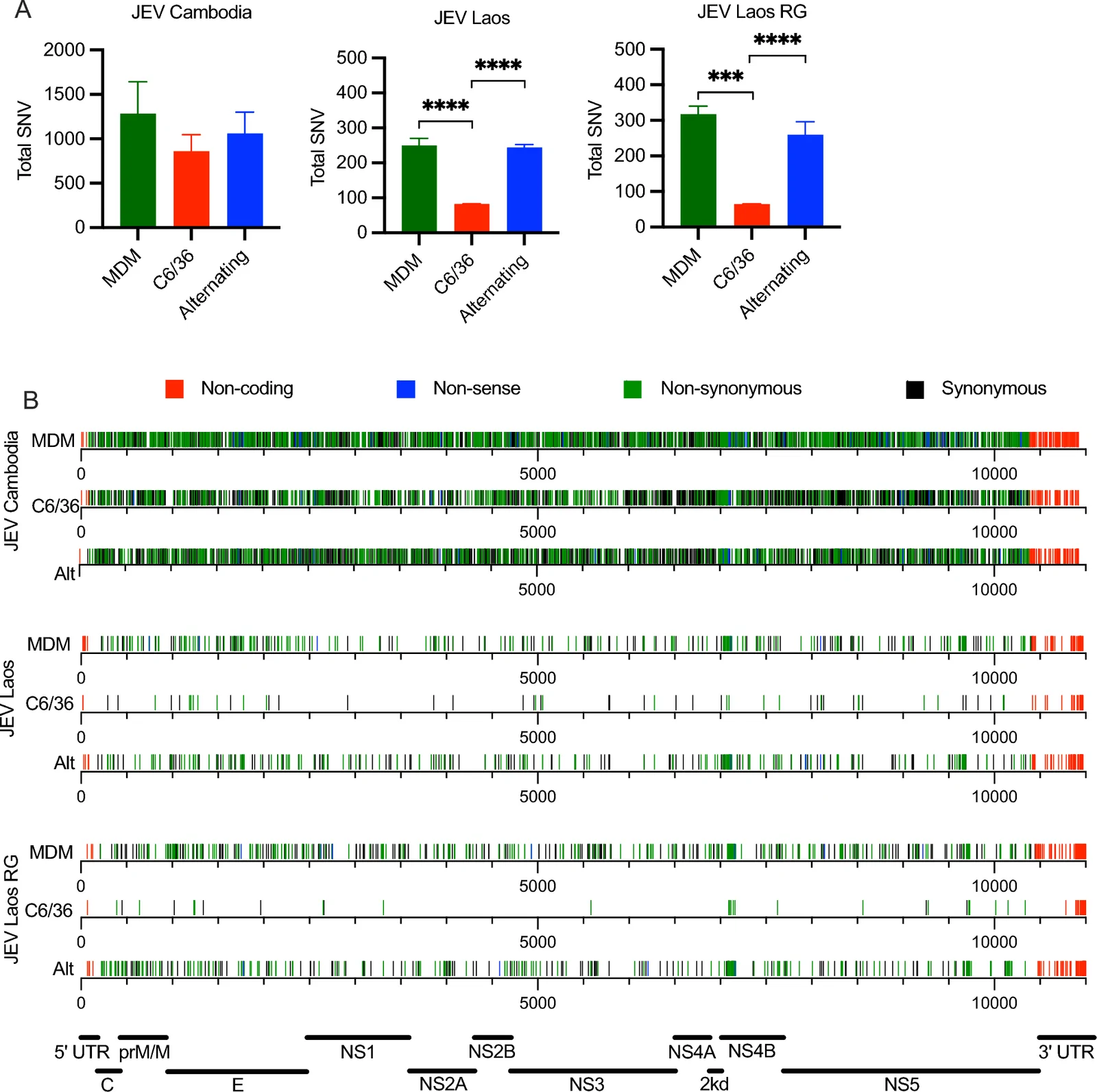
Total numbers of SNVs during passaging. In **A**, the total SNVs in all passages are shown, comparing their number in MDM, C6/36 and the alternating passaging for JEV Cambodia, Laos and Laos RG. Significant differences between the passaging were evaluated using Tukey’s multiple comparison test (ANOVA). Significance was depicted with *p<0.05, **p<0.01, ***p<0.001 and ****p<0.0001. In **B**, the locations and types of the SNVs are schematically represented by lines at their position in the genome, separating non-coding, non-sense, non-synonymous and synonymous SNVs by the colours

To determine the impact of cell type-specific JEV passaging on the viral genome, we identified the single nucleotide variants (SNVs) after passages P1, P3, P5, P7, P10 and P12 compared with the major alleles of the corresponding viruses in P0. For JEV Cambodia we collectively found 850 to 1250 SNVs, without any statistically significant differences between the different types of passaging (Fig. 4A). The number of identified variants for JEV Laos and JEV Laos RG were lower, with approximately 50 to 300 SNVs. Interestingly, for both JEV Laos viruses, C6/36 passaging caused reduced nucleotide divergence when compared to MDM or alternating passaging (Fig. 4A). Considering that JEV Laos RG was derived from a cDNA clone of JEV Laos, the similar level of nucleotide diversity of the two virus preparations was surprising and indicates that nucleotide diversity was rapidly re-acquired during the two passages in PEDSV.15 cells, which were needed to recover the cloned virus from cDNA and generate the P0 virus stock. As expected, the SNVs were evenly distributed across the genomes of all three viruses (Fig. 4B).

### Selection pressure on SNVs for JEV Cambodia

**Fig. 5:**
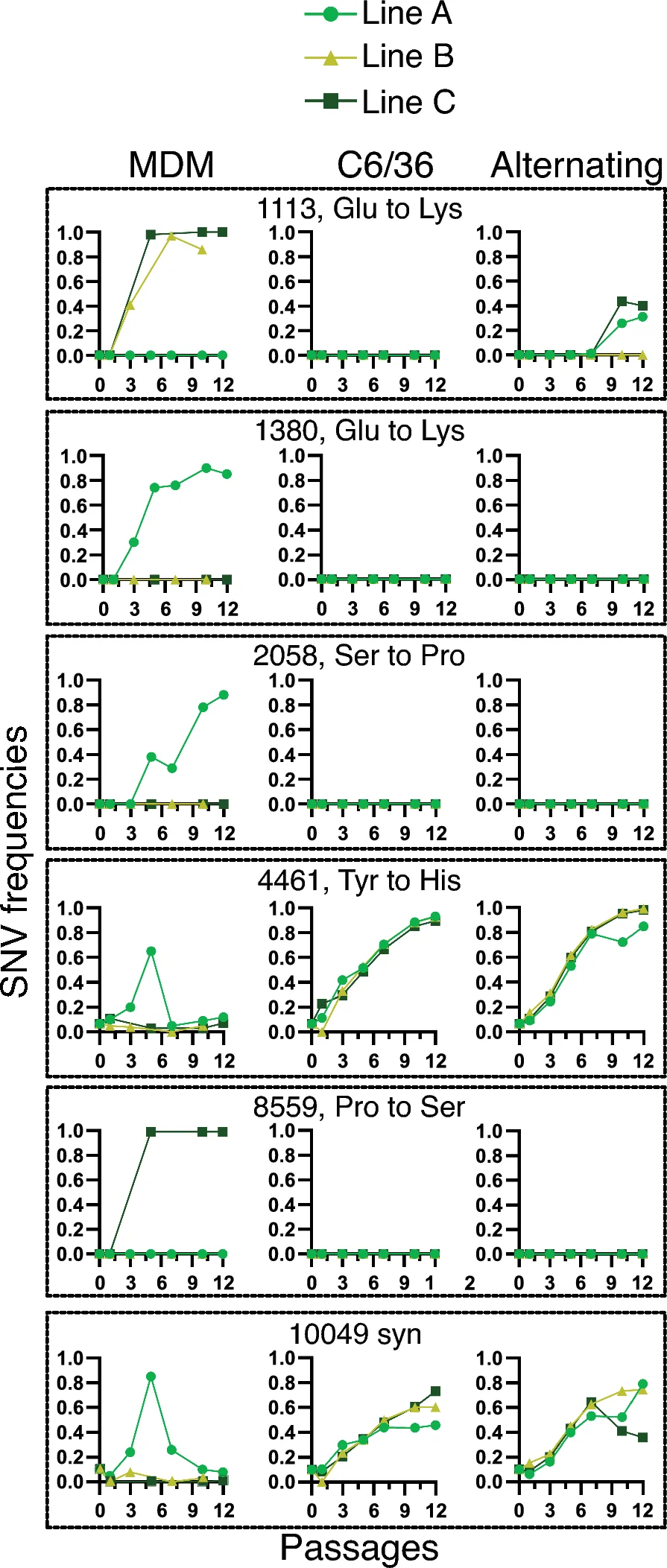
Trajectories of SNVs for JEV Cambodia. The figure shows frequencies for SNVs divergent from P0 that reached >50% at P12, in at least one line of passaging and at least in one cell culture condition. Frequencies (y-axis) for each line (**A**, **B** and **C**) are shown separately during the 12 passages (x-axis). The title of each plot indicates the locations in the genome, the effect on the amino acid sequence (syn=synonymous) and the passage of first SNV detection

To identify SNVs with selective advantage, we visualized SNV frequency development during the passages. Only SNV frequencies over 50% at any time during passaging were considered. For JEV Cambodia, six nucleotide positions causing five amino acid changes fulfilled these criteria (Fig. 5). The MDM passaging caused selection of four SNVs on positions 1113, 1380, 2058 and 8559. The position 1113 was seen in the two replica lines B and C, position 8559 was selected by line C, while 1380 and 2058 were only observed in line A. The fact that these variants were only detected first between P3 and P5 and not in all lines indicate that they may represent selected de-novo mutations. The trajectories demonstrate that the C6/36 and the alternating passaging caused quasi-identical selections of two pre-existing variants (positions 4461 and 10049) in all replicas during passaging.

### Selection pressure on SNVs for JEV Laos

**Fig. 6:**
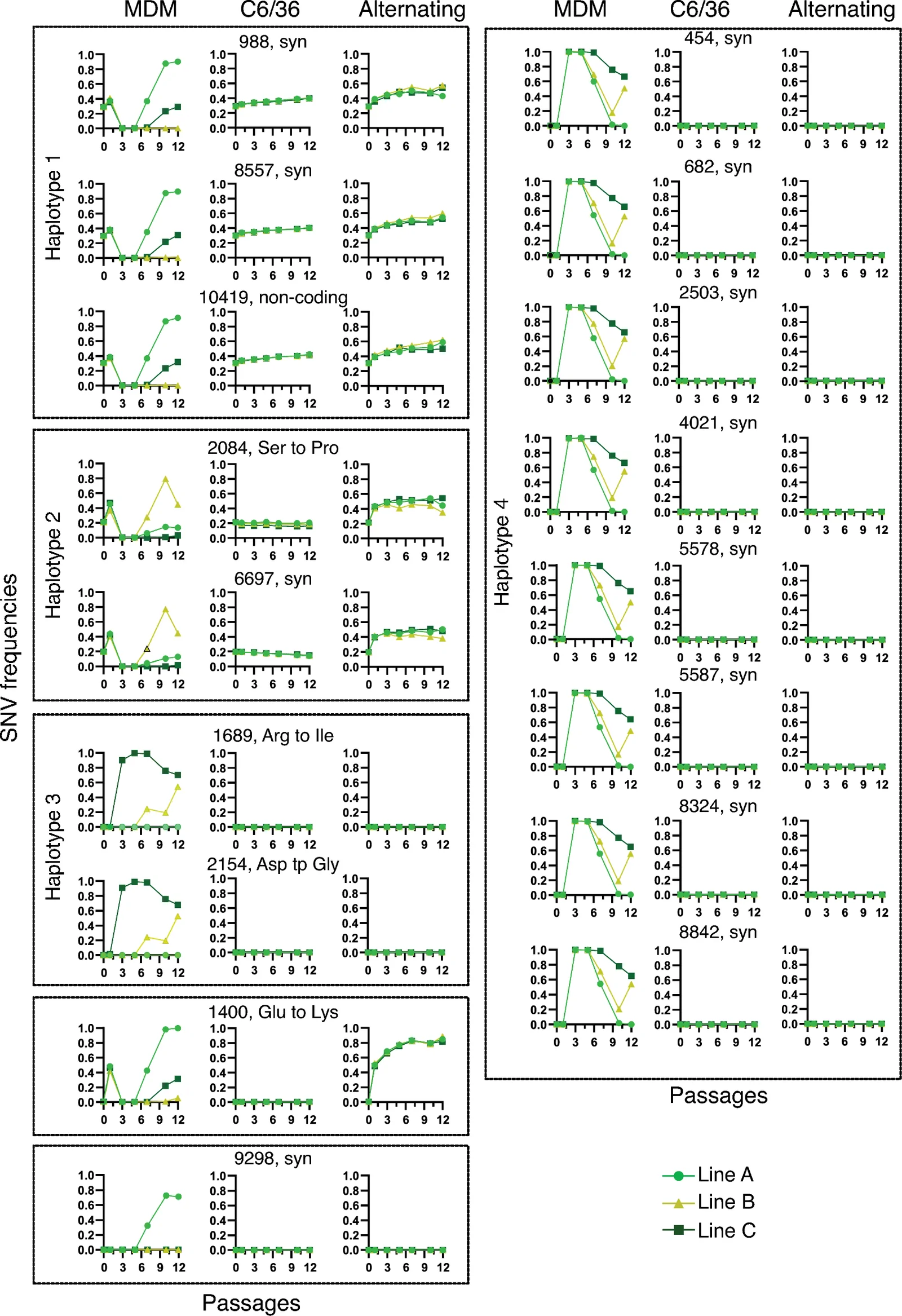
Trajectories of SNVs for JEV Laos. The frequencies of SNVs divergent from P0 that reached >50% at P12, in at least one line of passaging and at least in one cell culture condition are shown. The title of each plot indicates the location of the SNV in the genome and the effect on the amino acid sequence. Some SNVs are grouped into haplotypes (1-4) that were defined as two or more SNVs that showed quasi-identical frequencies (cosine similarity >0.99) during all passages

Using the same filter as for JEV Cambodia demonstrated that passaging of JEV Laos caused a selection of a total of 17 SNVs, with 12 being synonymous, four non-synonymous and one being in the non-coding region (Fig. 6). Overall, except for the position 1400, the selections were again similar for the C6/36 and the alternating passaging. With JEV Laos, the trajectories found in the three replica lines of MDM passaging permitted the identification of four viral haplotypes. These were defined when at least two SNVs showed quasi-identical trajectories along the lines of passages (Cosine similarities >0.99). Considering that we used short reads, we do not have conclusive evidence that alternate alleles for these variants are indeed located on the same haplotypes, but the pattern observed was best explained with haplotype selection in the MDM passaging. While haplotype 1 and 2 represented selected major variants with frequencies over 20% in P0, haplotypes 3 and 4 were not detected in P0. Nevertheless, haplotype 4 must represent a rare pre-existing haplotype considering that eight nucleotide positions had quasi-identical trajectories. None of these haplotypes appeared to be under strong selection pressure as they were not selected in all lines and showed fluctuating trajectories (Fig. 6). The only two SNVs selected in the MDM passaging were at positions 1400 (pre-existing variant) and 9298 (de-novo mutation).

### Selection pressure on SNVs for JEV Laos RG

**Fig. 7:**
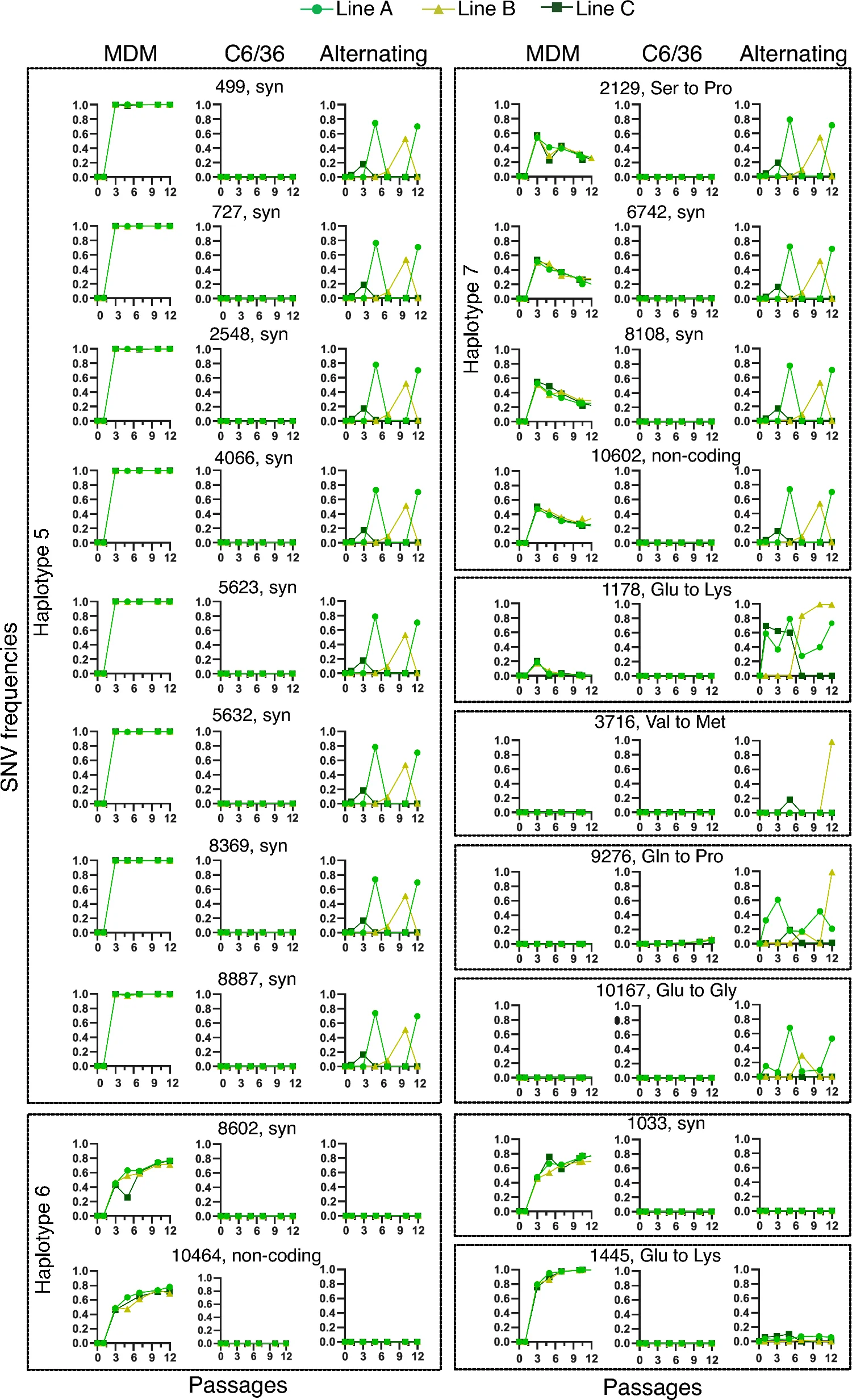
Trajectories of SNVs for JEV Laos RG. The frequencies of SNVs divergent from P0 that reached >50% at P12, in at least one line of passaging and at least in one cell culture condition are shown. The title of each plot indicates the location of the SNV in the genome and the effect on the amino acid sequence. Some SNVs are grouped into haplotypes (5-7) that were defined as two or more SNVs that showed quasi-identical frequencies (cosine similarity >0.99) during all passages

For JEV Laos RG, a total of 20 SNVs were selected (12 synonymous, 6 non-synonymous and 2 non-coding). The analysis of the trajectories for similarities identified three putative haplotypes and six independent SNVs (Fig. 7). Haplotype 5 with eight SNVs, as well as haplotype 6 with two SNVs were strongly selected by passaging in MDM. In the alternating passaging, we identified mainly quasi-identical trajectories of haplotypes 5 and 7 which showed a fluctuating behavior, reflecting the changing selection pressures from the alternating passaging. Nevertheless, as these two haplotypes clearly differed in the MDM passages, they cannot be identical. A possible explanation for the emergence of these two haplotypes would be two recombination events between a parent genome containing all nucleotide variants and another genome carrying the consensus sequences. The alternating passaging selected for the SNVs at positions 3716 and 9276 in replica line B, for position 10167 in line A and for position 1178 in lines A and B. The MDM passaging selected SNVs at position 1033 and 1445 in all three replica lines.

### Genotype to phenotype investigations

**Fig. 8:**
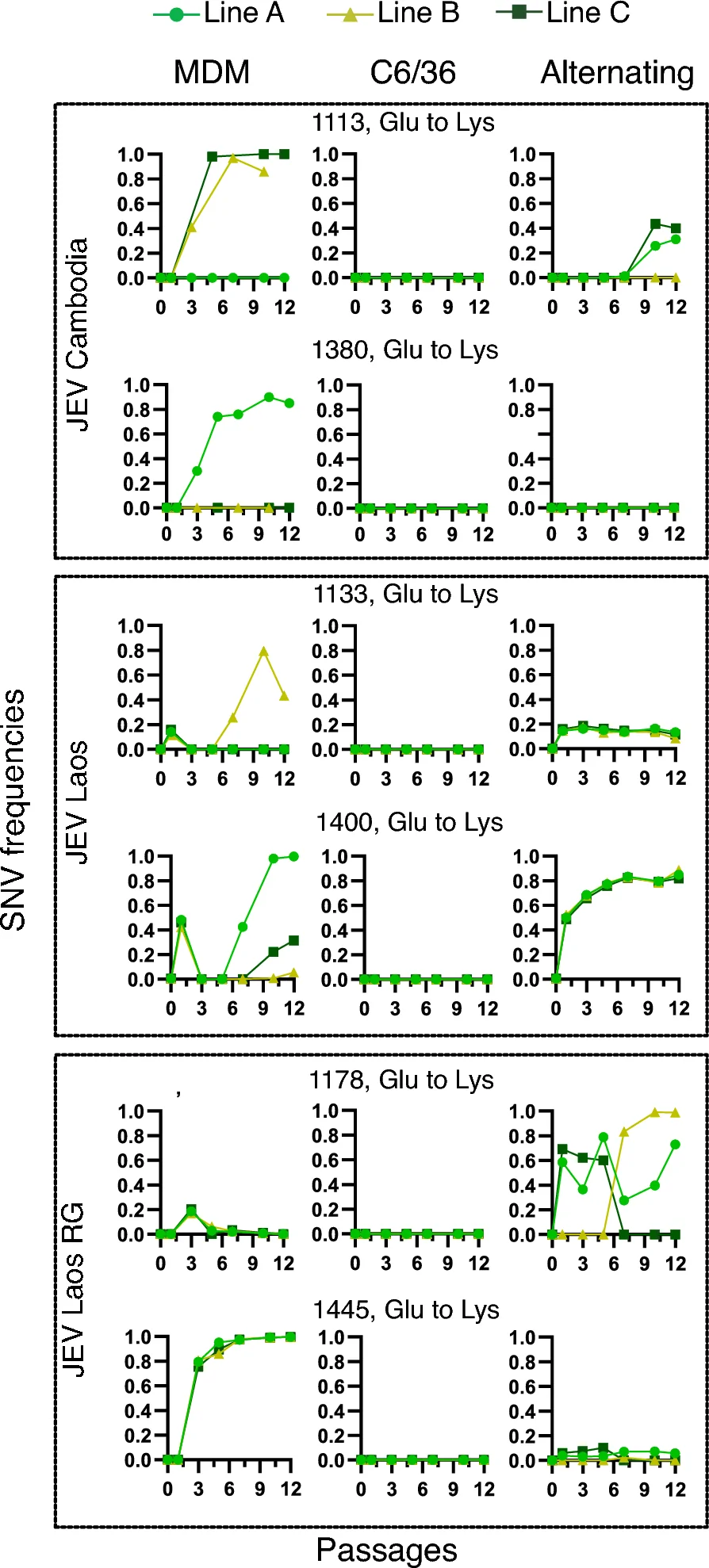
Selection pressure for E49K and E139K coding SNV. The data was filtered for SNV that reached >50% frequency at P12 and that caused identical amino acid changes in at least one of the lines for all three viruses. Two sets of mutations causing amino acid changes in the E protein were identified: (i) E49K mutation located in positions 1113, 1133 or 1178 for JEV Cambodia, Laos and Laos RG, respectively; (ii) E138K mutations located in positions 1380, 1400 or 11445 for JEV Cambodia, Laos and Laos RG, respectively. The Figure depicts the frequencies of these SNV during passaging

Considering that the major phenotypic changes induced by MDM passaging were found with all three JEV viruses tested, we searched the data for SNVs selected to high frequencies in at least one line of all the tested viruses. This analysis identified two nucleotide variants that induced a change in the amino acid sequence of the E protein, from glutamic acid (E) to lysine (K) at positions 49 and 138 (Fig. 8). The SNVs at positions 1113, 1133 or 1178 represented E to K substitutions at position 49 for JEV Cambodia, Laos and Laos RG, respectively. This SNV was selected in line B and C of JEV Cambodia and in line B of JEV Laos. The second set of SNVs were found at positions 1380, 1400 or 1445, causing a E to K change at position 138 of the E protein for JEV Cambodia, Laos and Laos RG, respectively. This substitution was selected in line A of JEV Cambodia, line A of JEV Laos and all lines for JEV RG-Laos. In two cases these mutations were also selected during the alternating passaging (SNV 1178 in line B of JEV RG-Laos and for SNV 1400 in all lines of JEV Laos).

**Fig. 9:**
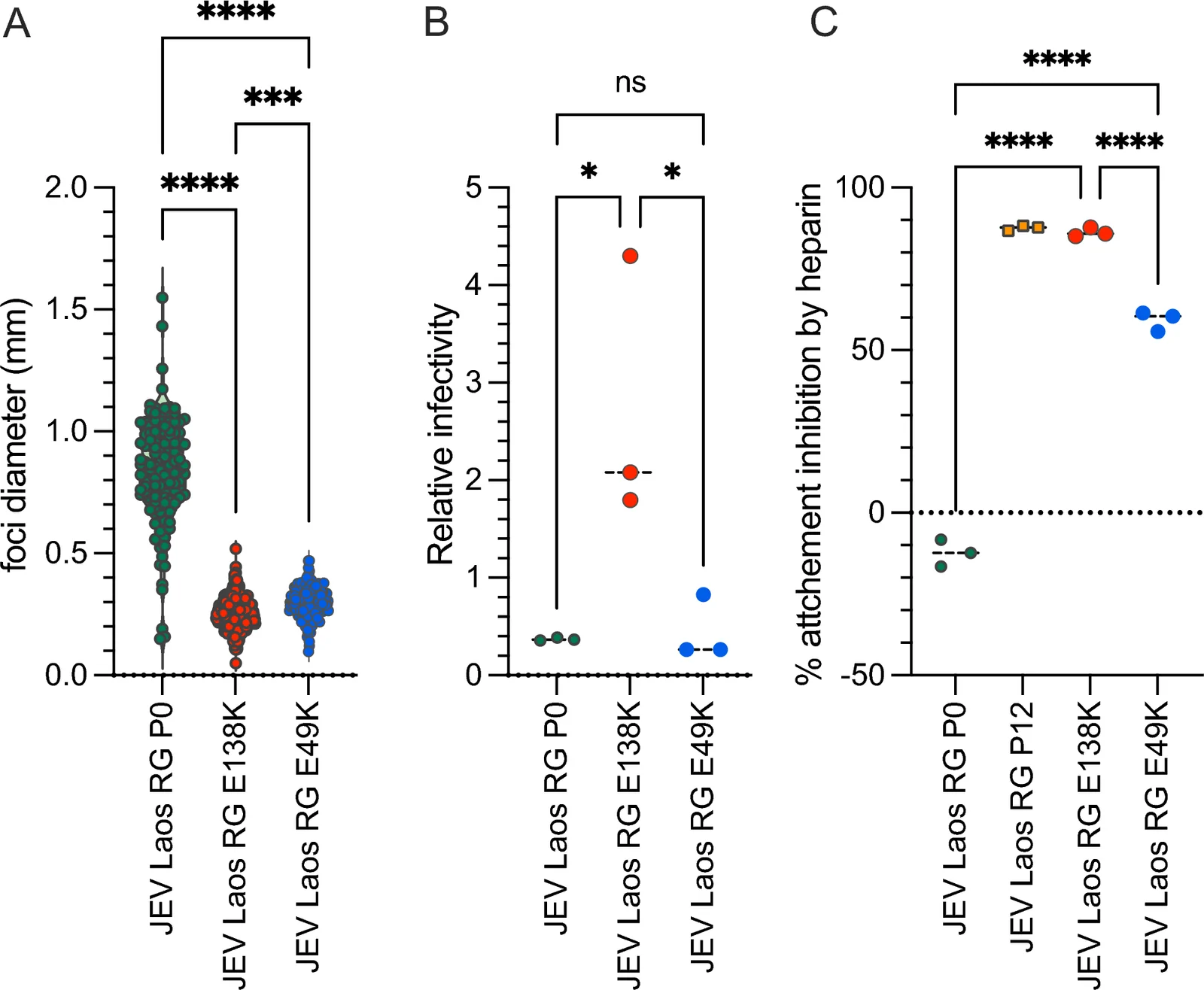
E49K and E138K explain the cell culture phenotype and cause heparin sensitivity. Using the reverse genetics, we introduced either the E49K or the E138K mutation in the JEV Laos RG and compared JEV Laos RG, JEV Laos RG E49K, JEV Laos RG E138K for foci size phenotype (**A**), relative infectivity determined as in Fig. 1 (**B**), and sensitivity of virus attachment inhibition by heparin (**C**). For C, we included the three lines of JEV Laos RG P12, which all acquired the E138K mutation by selection in MDM cultures

These analyses support an important role for E49K and E138K and indicate that either the E49K or the E138K mutation is necessary for the phenotype acquisition. To confirm this, we constructed JEV Laos RG clones that expressed either the E49K or the E138K. Indeed, as demonstrated in Fig. 9A, these clones uniquely displayed a small foci phenotype. Interestingly, the E138K viruses caused significantly smaller foci sizes than the E49K JEV. Only E138K but not E49K mutation caused increased relative infectivity for MDM when compared to the original JEV Laos RG (Fig. 9B). Considering that both positions were previously shown to mediate enhanced binding of JEV to GAG [18, 47], we performed virus attachment inhibition experiments using heparin known to efficiently inhibit GAG binding of JEV [18]. As expected, heparin efficiently inhibited attachment of JEV Laos RG P12, E138K and E49K mutants, but not JEV Laos RG P0. Interestingly, the E49K mutant was less sensitive to heparin inhibition when compared to the E138K variant, indicating a reduced binding to GAGs expressed on the cell membrane (Fig. 9C**)**.

## Discussion

The present study demonstrated that passaging of JEV in MDMs but not in C6/36 cells rapidly induced an increased relative infectivity for MDMs and a small-foci phenotype. The impact of alternating passaging was different between the viruses. While no phenotypic adaptations were found with JEV Cambodia, passaging JEV Laos and JEV Laos RG caused a mixed phenotype partially resembling that of MDM passaging. The high correlation of the increased relative infectivity for MDMs and the small foci indicate that these phenotypes might be caused by similar or linked genetic adaptations. Indeed, next generation sequencing demonstrated a higher selective pressure resulting in more SNV and haplotype selection when passaging in MDM as compared to C6/36 cells.

When comparing our data with published studies, there are conflicting reports with respect to the selective pressure in insects. West Nile virus (WNV) and St. Louis encephalitis virus showed increased replication after passaging in C6/36 cells [11]. Yet, SNV analysis after *in vivo* passaging of WNV in Culex pipiens mosquitos revealed no significant selection of SNVs, which is more in line with our analysis [10]. However, when JEV Nakayama was passaged on C6/36 cells, Vero cells, eggs or mice, the C6/36-passaged JEV selected for a similar number of SNVs than with Vero passaging, and a higher amount of SNVs when compared to passaging in eggs or mice [24]. It appears that the number of selected SNVs depends on the experimental set-up and the virus strain used in the assays. The absence of a pronounced adaptation towards the C6/36 cells in our experimentations could point to a high level of adaptation of our JEV strain to the vector. This was also proposed for St. Louis encephalitis virus as passaging in mosquitos did not result in fitness gain in insects [9]. Nevertheless, it should be noted that C6/36 cells might not be the best model to answer this question. First, the cells are derived from *Aedes* mosquitos, which is not the main vector for JEV in nature. Second, C6/36 cells have defects in the RNAi pathway, that has been shown to impact quasispecies distribution for WNV [6, 7, 35]. The defective RNAi pathway needs to be taken into consideration when interpreting the observed genetic stability of JEV in C6/36 cells. It is possible that passaging in insect cells with fully functional RNAi pathways would cause a higher adaptive pressure. Therefore, future studies using *in vivo* passaging of JEV in *Culex* mosquitos might give a more valuable insights on insect-specific viral adaptations.

We also tested how alternating passaging impacted virus adaptations. Our data indicates that the selective pressure resembles more that of only insect cell passaging. This was seen for JEV Cambodia and JEV Laos, in which an identical selection of SNV was observed, except for a selective pressure on the SNV that cause the E138K and E49K changes in the E protein. For JEV Laos RG, the alternating selection pressures caused fluctuations of two haplotypes. This indicates that viral variants selected in MDM loose fitness in C6/36 cells, and therefore the follow-up passage in C6/36 cells, can partly reverse the selection in MDM. This supports the concept that the natural alternating cycling between mosquitos and vertebrates can limit species adaptation. Our data is in line with a study that passaged Dengue virus on human Huh-7 cells, on C6/36 cells or in an alternating manner. Human cell passaging caused a more rapid and increased accumulation of SNVs and showed increased fitness in the cell type of passaging [40].

For JEV Cambodia, our passaging also selected the SNV at position 4461 resulting in a Y86H change in the NS2b protein following C6/36 and alternating passaging. Interestingly, the histidine on position 86 was reported for several JEV isolates (e.g. GenBank: LC461958; KY927815.1; OM572543.1; JF706282.1), as well for JEV Laos (KC196115).

A main finding of our data was that with all three viruses, MDM cultures caused a major selective pressure favoring JEV with the amino acid changes E49K or E138K in the E protein, which favored GAG binding. GAG are abundantly expressed on mammalian compared to insect cells [1], providing a possible explanation why the fast adaptation was mainly observed for the MDM passaging. For the JEV Laos strain, the E138K mutation was associated with the highest heparin sensitivity, and explained both increased infectivity for MDM and the small foci size phenotype. In contrast, the E49K mutant was found to be less sensitive to heparin inhibition, which may explain its higher plaque size and the low relative infectivity for MDM. This would indicate that an increase in relative infectivity for MDM requires a strong enhancement of GAG binding.

An explanation for the small plaque phenotype is that high GAG binding tethers viruses on the cell membrane after exocytosis and thereby limits cell-to-cell spread, which represents the main mode of viral expansion in the cell cultures forming monolayers that are kept in semisolid medium. In contrast, cell-to-cell spread is not involved in MDM cultures which do not form monolayers. Therefore, the increased relative infectivity for MDM of the E138K mutants may have been caused by increased binding of MDM, representing a selective advantage in these cultures.

Identical adaptations were previously demonstrated to be also induced in hamster kidney or human SW13 cells, and to be also associated with a small plaque phenotype as well as increased binding to GAGs [17, 47]. Interestingly, line C of MDM-passaged JEV Laos was the only line that neither acquired E49K nor E138K at high frequencies but instead a D389G change (position 2154 in the genome). Also this amino acid change was previously associated with increased GAG binding by JEV [17, 18].

Importantly, amino acid changes associated with increased GAG binding are not selected when JEV Laos was passaged in pigs [22], indicating that the selection processes *in vivo* differ from those *in vitro* despite the use of primary macrophage cultures. Furthermore, such adaptations typically result in attenuation of *Orthoflaviviruses* [2]. For instance, E138K was shown to relate to the attenuation of the JEV vaccine strain SA14-14-2 reducing its neurovirulence [16, 21, 26, 46]. While increased attachment to cells is beneficial for the virus when cultured in certain cell types, the tethering of exocytosed viruses to the cell membrane might limit viremia and viral spread *in vivo,* which would explain why such viral variants are not selected in the field [2].

## Conclusions

Serial passaging of JEV in porcine macrophages imposed strong evolutionary pressure, leading to rapid phenotypic and genetic adaptations, whereas insect cells exerted comparatively limited selective pressure. Alternating passaging partially constrained this selective pressure, reflecting the balancing selection expected during natural dual-host transmission cycles. Thus, the data supports previous observations that single host cycling can rapidly reshape viral quasispecies composition. However, the consistent selection of envelope mutants with enhanced GAG binding appears to be driven by *in vitro*-specific selective pressures, underscoring the importance of carefully choosing propagation systems and monitoring virus stocks for culture-adaptive mutations.

## Supplementary Information

Additional file 1.

Additional file 2.

## Data Availability

Data generated during this study is available in supplementary material.
