# Supplementary material for: Comparative dynamics of Japanese encephalitis virus adaptation in porcine macrophages and insect cells
Source: Virol J. 2026 Jun 27;23:210. doi: 10.1186/s12985-026-03231-0 (PMC13573486; doi:10.1186/s12985-026-03231-0)
Supplement: Supplementary file 1 [file 12985_2026_3231_MOESM2_ESM.docx]

**Supplementary Data**

**Supplementary Figure 1. Changes in foci sizes in C6/36 cultures following passaging of JEV in MDM.** JEV Cambodia (A), JEV Laos (B) and JEV Laos RG (C) were passaged 10 times in MDM, C6/36, or in both cell types in an alternating manner (left to right). At the indicated passages the viruses were characterized by focus-forming assays. Line A is depicted in blue, line B in red and line C in green.

**Supplementary Figure 2.** **Stability of the small-foci size phenotype during reverse passaging in C6/36 cells**. P10-MDM JEV (blue) of JEV Cambodia, Laos and Laos RG were passaged an additional five times on insect C6/36 cells (red), and the P10, P11 and P15 viruses were compared in a focus forming assay using C6/36 and PEDSV.15 cells. All foci sizes were compared to P0 by the Tukey’s multiple comparison test (*p<0.05, **p<0.01, ***p<0.001 and ****p<0.0001).

**Supplementary Table 1.** Statistical analyses of plaques size data from Figure 2. 2-way Anova and Dunnett’s correction for multiple comparisons was employed to compare plaque sizes within each line of passaging to plaques sizes before passaging (“0”).
